# Shading contributes to *Sphagnum* decline in response to warming

**DOI:** 10.1002/ece3.10542

**Published:** 2023-09-19

**Authors:** Richard J. Norby, Taylor Baxter, Tatjana Živković, David J. Weston

**Affiliations:** ^1^ Environmental Sciences Division Oak Ridge National Laboratory Oak Ridge Tennessee USA; ^2^ Department of Ecology and Evolutionary Biology University of Tennessee Knoxville Tennessee USA; ^3^ Department of Biology Dalhousie University Halifax Nova Scotia Canada; ^4^ Biological Sciences Division Oak Ridge National Laboratory Oak Ridge Tennessee USA

**Keywords:** bog, climate change, peatland, shrubs, *Sphagnum angustifolium*, *Sphagnum divinum*, *Sphagnum fallax*, warming

## Abstract

Experimental warming of an ombrotrophic bog in northern Minnesota has caused a rapid decline in the productivity and areal cover of *Sphagnum* mosses, affecting whole‐ecosystem carbon balance and biogeochemistry. Direct effects of elevated temperature and the attendant drying are most likely the primary cause of the effects on *Sphagnum*, but there may also be responses to the increased shading from shrubs, which increased with increasing temperature. To evaluate the independent effects of reduction in light availability and deposition of shrub litter on *Sphagnum* productivity, small plots with shrubs removed were laid out adjacent to the warming experiment on hummocks and hollows in three blocks and with five levels of shading. Four plots were covered with neutral density shade cloth to simulate shading from shrubs of 30%–90% reduction in light; one plot was left open. Growth of *Sphagnum angustifolium/fallax* and *S. divinum* declined linearly with increasing shade in hollows, but there was no response to shade on hummocks, where higher irradiance in the open plots may have been inhibitory. Shading caused etiolation of *Sphagnum*—they were thin and spindly under the deepest shade. A dense mat of shrub litter, corresponding to the amount of shrub litter produced in response to warming, did not inhibit *Sphagnum* growth or cause increases in potentially toxic base cations. CO_2_ exchange and chlorophyll‐*a* fluorescence of *S. angustifolium/fallax* from the 30% and 90% shade cloth plots were measured in the laboratory. Light response curves indicate that maximal light saturated photosynthesis was 42% greater for *S. angustifolium/fallax* grown under 30% shade cloth relative to plants grown under 90% shade cloth. The response of *Sphagnum* growth in response to increasing shade is consistent with the hypothesis that increased shade resulting from shrub expansion in response to experimental warming contributed to reduced *Sphagnum* growth.

## Graphical abstract

*Sphagnum* was maintained under shade cloth of different densities to simulate the effect of shading from shrubs in SPRUCE enclosures. *Sphagnum* production declined with increasing shade, supporting the hypothesis that *Sphagnum* decline in response to warming was due in part to increased shade from shrubs.

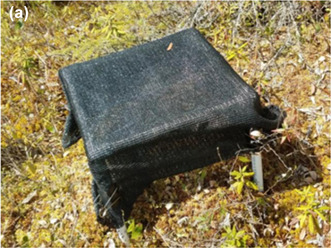

## 1 INTRODUCTION

Boreal peatlands contain a vast store of carbon (C) that has accumulated over centuries and millennia, and those carbon stocks are especially vulnerable to climate change (He et al., 2016; Wilson et al., 2016). Warming and associated drying of the peat will stimulate respiration and decomposition, processes that have been retarded because of the cold, wet conditions characteristic of bogs and other peatlands, potentially converting this critical biome from a net sink of global C to a net source (Gallego‐Sala et al., 2018; Hanson et al., 2020). The resulting release of C as CO_2_ or CH_4_ to the atmosphere represents an important positive feedback, exacerbating climatic warming (Moore et al., 1998). The past and current source of much of the C in peatlands are mosses of the genus *Sphagnum*. *Sphagnum*, as an “ecosystem engineer” with its unique adaptations to low nutrient availability and a chemical composition that resists decomposition, helps to perpetuate the bog environment by maintaining acidic conditions and limiting competition from co‐occurring vascular species (van Breemen, 1995). External factors, such as climatic warming or increased nitrogen (N) availability (fertilization or deposition), can alter the competitive balance and compromise the role of *Sphagnum* in ecosystem functioning (Bubier et al., 2007; Ma et al., 2022).

**FIGURE 1:**
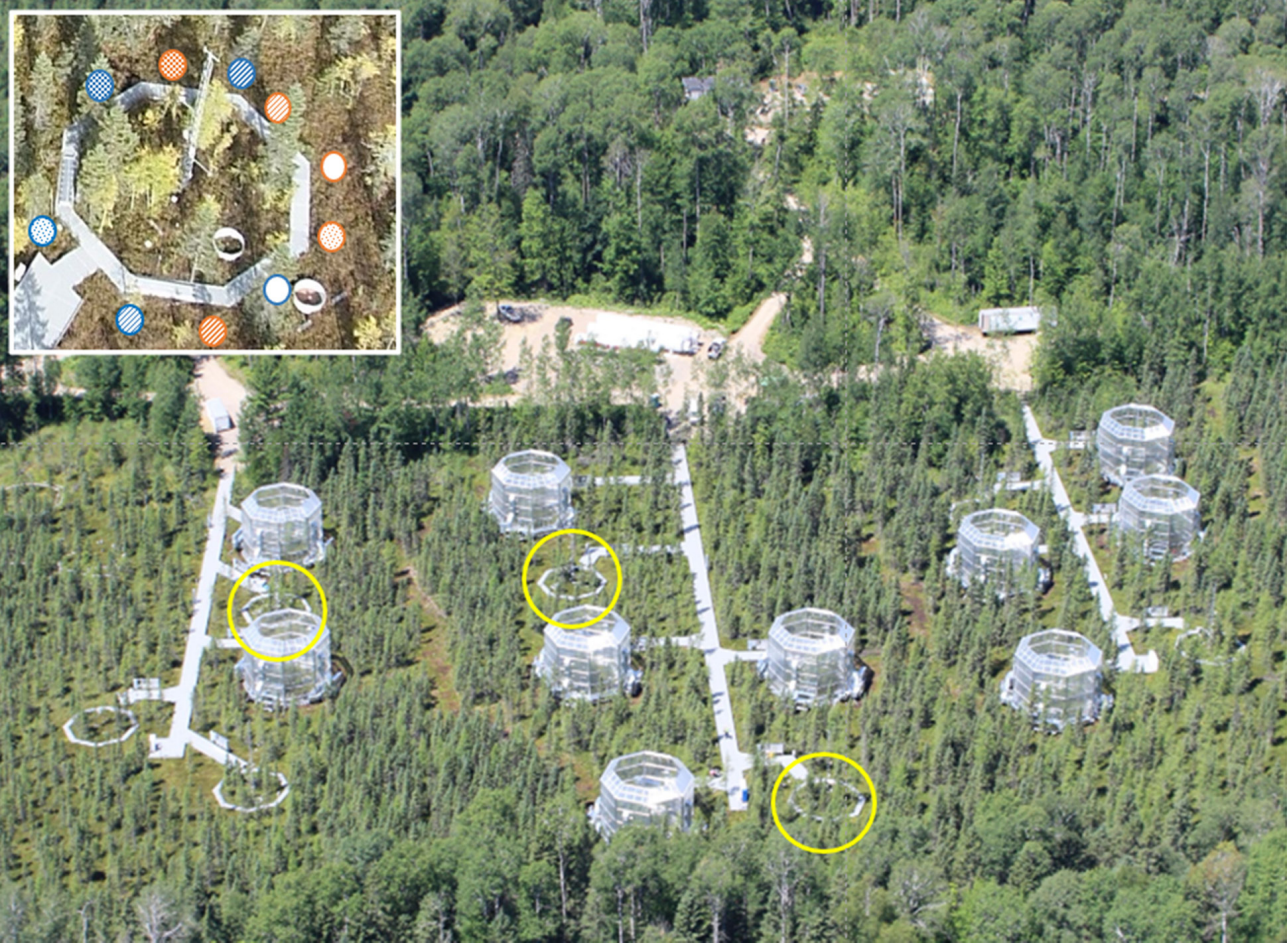
Aerial view of the S1 bog in the Marcel Experimental Forest, showing the three locations (blocks) of the shade experiment (yellow circles), which were interspersed among 10 enclosures used in the SPRUCE warming × CO_2_ experiment. Inset: Layout of 10 shade treatments in one block. Blue circles are hollow locations, and orange circles are hummocks. Shade level (0%, 30%, 60%, 80%, 90%) is indicated by the density of cross‐hatching. Photo reference: Hanson et al., 2015.

Here, we investigate the responses of a *Sphagnum* community to reduction in availability of light. The questions we ask, and the approach we took, were motivated, and informed, by the response of an ombrotrophic bog to experimental warming and CO_2_ enrichment in the SPRUCE experiment (Spruce and Peatland Responses Under Changing Environments, https://mnspruce.ornl.gov/), (Hanson et al., 2020). The climate change manipulations in the SPRUCE experiment were delivered in large, octagonal, open‐top enclosures (Figure 1) and comprised air and peat warming levels of +0, +2.25, +4.5, +6.75, and +9°C in ambient and elevated CO_2_ (ambient +500 ppm; Appendix S1). There were strong effects of the experimental treatments on the *Sphagnum* community (Norby et al., 2019). The response of *Sphagnum* productivity to warming and CO_2_ enrichment in 2019–2021 (Figure S1a) was very similar to that observed previously. NPP declined linearly with increasing temperature, and there was a significant interaction with CO_2_ such that NPP was lower in elevated CO_2_ in the cooler enclosures. There was little response to the treatments in the first year of exposure (2016), but the response became apparent in the second and third years, with little change after that (Figure S1b,c). The loss of cover was the primary contributor to the decline in NPP (Norby et al., 2019; Petro et al., 2023).

These effects of warming, and to a lesser extent CO_2_, on *Sphagnum* productivity in the SPRUCE experiment have larger scale implications for the structure and function on the bog ecosystem (Hanson et al., 2020; Iversen et al., 2022). Hence, it is especially important to delineate the environmental drivers controlling the responses, which should help to inform modeling of *Sphagnum* dynamics in the face of climate change (Shi et al., 2021). Our previous analysis (Norby et al., 2019) suggested direct effects of temperature and the attendant drying on productivity, but the basis of the negative response to elevated CO_2_ was not clear. In addition to these direct environmental drivers, there may be indirect effects derived from the increase in shrub cover with warming that has been documented in the SPRUCE experiment. McPartland et al. (2019) measured Normalized Difference Vegetation Index (NDVI) of the understory plant community—a proxy for shrub cover—within the SPRUCE enclosures. There was a significant effect of temperature and an interaction between CO_2_ and temperature, with NDVI greatest in the warmest chamber, indicating that warming had caused an increase in shrub cover. Subsequent direct measurement of leaf area and monitoring of the composition of the vascular plant community revealed different responses of the several shrub species and an overall increase in LAI with warming (McPartland et al., 2020). The dense shrub layer in the warmer enclosures was visually quite apparent (Figure S2a); it was much less dense in the cooler enclosures. Shrubs could affect *Sphagnum* productivity through competition for water and nutrients, through shading and reduction in light and alteration of light quality to the *Sphagnum* layer, or through physical or chemical interactions with shrub leaf litter (Figure S2b). At the Mer Bleue bog in Canada, N fertilization stimulated shrub production, and it was inferred that shading by the shrubs led to a decline in *Sphagnum* productivity (Chong et al., 2012). In the intact bog in the current experiment, these potential influences on *Sphagnum* are confounded, and evaluation of their potential importance requires separating them. Hence, we set up an experiment to evaluate the effects of light reduction due to shading and shrub litter on *Sphagnum* productivity without any direct influence of other changes (e. g. water or nutrient supply) that the shrubs might impart.

Motivated by the observed responses of *Sphagnum* in the SPRUCE experiment. We evaluated several hypotheses:

1. *Sphagnum* productivity will decline beneath deep shade similar to the level of light reduction observed in the warmest SPRUCE chambers.
2. *Sphagnum* growth reductions under deep shade will be reflected in declines in photosynthesis.
3. Accumulation of leaf litter from shrubs on top of *Sphagnum* inhibits growth through physical effects (e.g., impact or light reduction) or through chemical interactions.

These hypotheses were evaluated in a replicated experiment established in 2021 in the same bog as the SPRUCE experiment, but outside and separate from the SPRUCE enclosures and the associated warming and CO_2_ treatments. Shrubs were removed from small plots to avoid any direct effects of shrubs, and the plots were covered with neutral density shade cloth to simulate the reduction in light availability created by shrubs. Shrub litter was added to subplots within the shade treatments. Photosynthetic responses were evaluated in the laboratory on stems harvested from the shade plots.

## 2 MATERIALS AND METHODS

### 2.1 Site description

The research site is an ombrotrophic bog in northern Minnesota, United States (47.50283° latitude, −93.48283° longitude). The bog is dominated by *Picea mariana* (Mill.) B.S.P. and *Larix laricina* (Du Roi) K. Koch trees, with an understory of ericaceous shrubs, including Labrador tea (*Rhododendron groenlandicum* (Oeder) Kron and Judd) and leatherleaf (*Chamaedaphne calyculata* (L.) Moench), and a limited number of herbaceous plants (Griffiths et al., 2017; Hanson et al., 2020). The bog has a hummock and hollow microtopography with shrubs located primarily (but not exclusively) on the hummocks. Prior to the onset of warming treatments, there were fewer shrub roots in hollows, but root density in hollows increased with warming and associated drying (Malhotra et al., 2020). Also, the scale of the hummock–hollow topography is such that shrubs on hummocks overhang and shade hollows. Across the bog (except in the warmest SPRUCE enclosures), there is a nearly continuous cover of mosses, primarily *Sphagnum angustifolium* (C.E.O. Jensen ex Russow) C.E.O. Jensen, *S. fallax* (Klinggr.) Klinggr., *and S. divinum* Flatberg & K.Hassel (previously called *S. magellanicum* Brid.). As in other similar bogs, *S. fallax* is found predominantly in hollows, whereas *S. angustifolium* and *S. divinum* are found predominantly in somewhat drier microhabitats, including lawns, low hummocks, and the flanks of high hummocks. *Sphagnum angustifolium* and *S. fallax* are difficult to distinguish in the field, and since they are closely related phylogenetically, we have not attempted to separate them in our analyses; we refer to them here *as S. angustifolium/fallax* (or *S. ang/fal* in figures).

### 2.2 Experimental design

**FIGURE 2:**
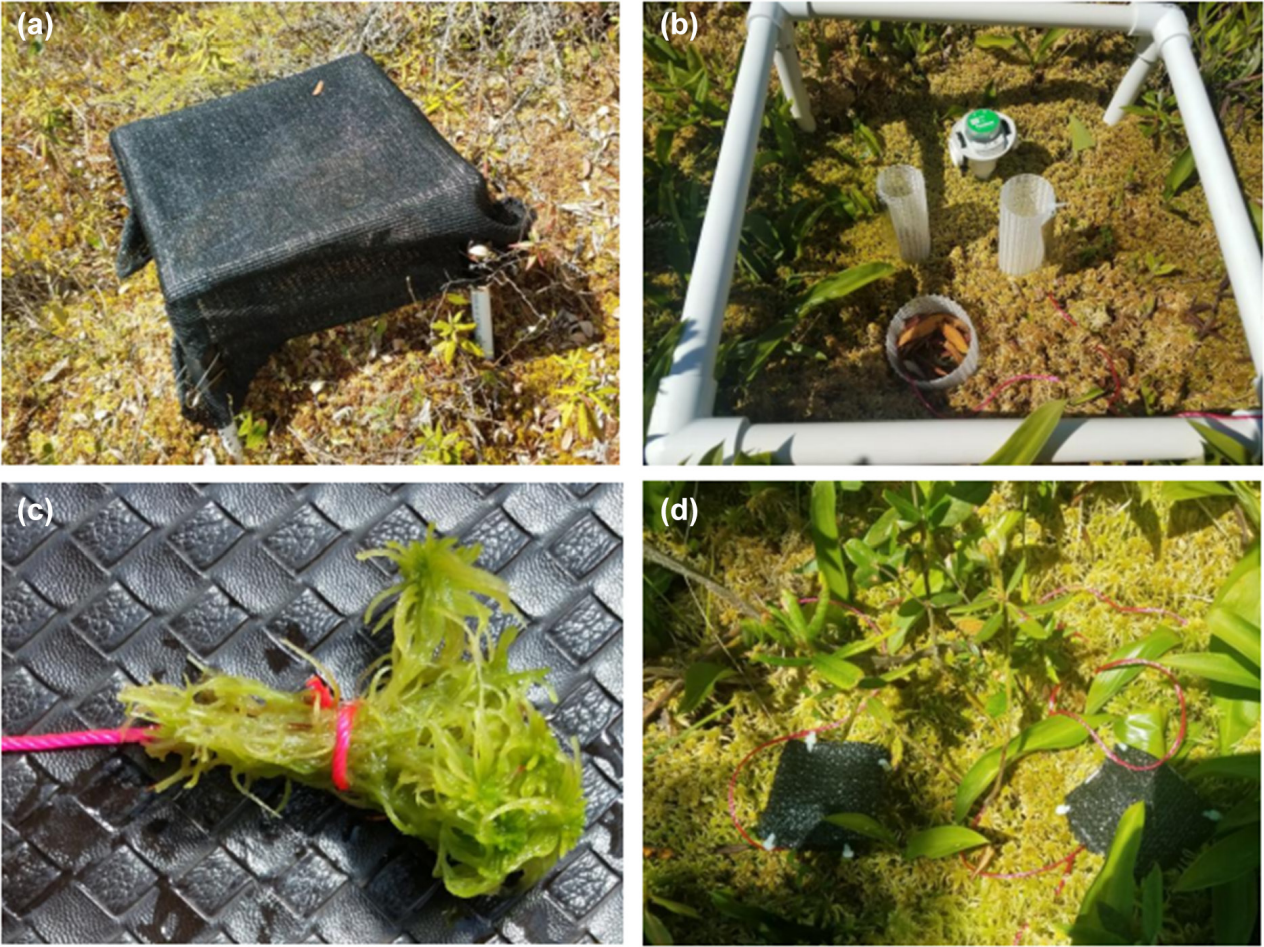
(a) Shade cloth deployment over a 35 × 35 cm frame. (b) Growth columns, litter addition, and HOBO for light measurements within an open shade treatment. (c) Bundle of 10.5‐cm long *Sphagnum* stems. (d) Small shade enclosures in hummock and hollow locations over *Sphagnum* bundles.

The experiment to test the response of *Sphagnum* to reduction in light availability was set up in a randomized complete block design. The three blocks were located adjacent to the SPRUCE experiment enclosures and included boardwalks and a central meteorological tower (Figure 1), but there had been no other manipulations. Ten 35 × 35 cm plots were established in each block (30 in total), five of which were on hummocks and five in hollows (Figure 1 inset). The plots were assigned to one of five levels of light reduction, nominally 0% (no shade cloth), 30%, 60%, 80%, and 90% shade. These levels were chosen to encompass the range of shading by shrubs in spot measurements made over both live and dead *Sphagnum* (Figure S3). Black shade cloth of the specified density (Greenhouse Megastore) was suspended approximately 10 cm over a plot, supported by a frame constructed from PVC pipe (Figure 2a). All shrubs and other vascular plants were clipped from within the plots, including adjacent shrubs that were overhanging the plot area. Any regrowth of shrubs was removed during the course of the experiment. The shade cloth treatments were initiated on May 17, 2021.

*Sphagnum* growth response to light reduction was assessed using the same approach as in the SPRUCE experiment (Norby et al., 2019). *Sphagnum angustifolium/fallax* and *S. divinum* were collected from a common area of the bog, separated by species and cleaned of debris. Twenty‐nine stems of *S. angustifolium/fallax* and 17 stems of *S. divinum* (corresponding to their stem densities in the bog) were cut to 5 cm in length and inserted into 38‐mm diameter plastic mesh columns. The top of the capitula was marked on the mesh, and the height relative to top of the column was recorded. The two columns were installed in each shade plot such that the top of the *Sphagnum* was at the same height and in good contact with surrounding *Sphagnum* (Figure 2b). The columns were adjusted periodically as necessary to maintain this connection with surrounding *Sphagnum*.

The actual light level depended on the shade cloth density and the overhead tree cover (shrubs had been removed.) We measured light at the *Sphagnum* surface using HOBO MX2202 data loggers (Onset) (Figure 2b). The HOBOs recorded light every 5 min; the data were downloaded and processed monthly. The HOBOS measured illuminance in units of lux, requiring calibration to μmol PAR m^−2^ s^−1^. Calibration was achieved by setting the HOBOS adjacent to a quantum sensor (LI‐COR 190R) under a range of sky conditions and under shade cloth. There was a linear relationship: PAR (μmol m^−2^ s^−1^) = 0.0209 × illuminance (lux). The 5‐min HOBO data were converted to PAR and summed to calculate 30‐min averaged PAR and daily PAR. Shade levels were calculated relative to the open plots. Other expressions of the light environment include average daytime (sunrise to sunset) PAR and PAR at solar noon (Table 1).

**TABLE 1:** Light conditions beneath shade cloth.

Topography	Nominal shade (%)	Daily total PAR (mol m^−2^ day^−1^)	% shade	Average daytime PAR (μmol m^−2^ s^−1^)	PAR at solar noon (μmol m^−2^ s^−1^)
Hummock	0	26.4 ± 2.5	0	513 ± 26	954 ± 67
Hummock	30	13.1 ± 1.3	50.5	254 ± 17	545 ± 33
Hummock	60	6.5 ± 0.9	75.3	127 ± 12	251 ± 41
Hummock	80	2.8 ± 0.5	89.5	76 ± 18	116 ± 8
Hummock	90	1.4 ± 0.3	94.8	28 ± 6	50 ± 15
Hollow	0	18.5 ± 1.3	0	359 ± 20	738 ± 97
Hollow	30	10.3 ± 2.1	44.2	200 ± 35	405 ± 63
Hollow	60	5.9 ± 0.6	68.3	114 ± 6	271 ± 23
Hollow	80	2.6 ± 0.2	85.7	46 ± 8	115 ± 24
Hollow	90	1.4 ± 0.3	92.6	26 ± 4	52 ± 7

*Note*: Nominal shade is that specified by the shade cloth manufacturer. Actual % shade is calculated relative to the open plots, and these are the values used for subsequent analyses. Average daytime PAR is the average of all values between sunrise and sunset, and PAR at solar noon is the average of all reading during the hour of solar noon. All data are the mean of three plots ± SE.

An additional 7.3 cm diameter mesh column was installed within each 35 × 35 cm plot to test the effects of shrub litter on *Sphagnum* nutrient composition. Senescent but still attached leaves were collected from *Rhododendrum* and *Chamadaphne*. The collection, comprising about 85% *Rhododendron* and 15% *Chamadaphne*, was well mixed, and 1.7 g fresh weight (1.5 g dry weight equivalent) was parceled out for each plot in the mesh columns (Figure 2b). This amount corresponds to the amount of litter accumulated in areas devoid of *Sphagnum* in +9°C SPRUCE enclosures, where shrub litter production was greatest (Figure S2b).

Aboveground shrub removal might have had an unspecified effect on *Sphagnum* other than the measured effects on light level. To test for this possibility, bundles of 10 5‐cm long *S. angustifolium* stems and 10 5‐cm long *S. divinum* stems were tied together with string (Figure 2c). Two bundles of each species were inserted into one hummock and one hollow location within each block; no shrubs were removed. A 5 x 5 cm square of 90% shade cloth, supported on a wire frame covered one bundle of each species, the others serving as controls (Figure 2d).

### 2.3 Measurements

*Sphagnum* height growth was measured biweekly in situ as the change in distance from column top to *Sphagnum* top, except no measurements were made in August because extreme drying of the *Sphagnum* during an extended drought made it too fragile to sample. *Sphagnum* moisture content was measured seven times during the year (not in August) on 10 stems collected from several locations within the plots and cut to 5 cm length. Fresh mass was measured immediately, and dry mass was measured after oven‐drying at 70°C. Moisture content was expressed as fresh mass minus dry mass divided by dry mass. Dry mass per unit length also was calculated. Similar samples were collected adjacent to each shade plots and their moisture content measured.

### 2.4 Harvest

The experiment was terminated on October 14. The columns were removed, and final height from *Sphagnum* top to column top was measured. The columns and the *Sphagnum* inside were cut at the mark where the initial capitula top was, and the new growth was weighed fresh, separated into stems and capitula, oven‐dried, and weighed. The *Sphagnum* in the litter columns was collected, the shrub litter was separated, and the *Sphagnum* and litter were oven‐dried. *Sphagnum* samples (0.5–1.0 g) from beneath the added litter were prepared for complete mineral analysis, including calcium (Ca) and magnesium (Mg), at the University of Georgia (EPA Method 3051 & 200.2). To obtain sufficient material, samples from hummock and hollow locations were combined, and in some cases, samples from the two or three blocks were combined. The bundles beneath the small shade shelters were collected, the bottom 5 cm of stem removed, and the length and dry mass of the new growth measured.

### 2.5 Light response curves

*Sphagnum* samples of *S. angustifolium/fallax* from hollows of the 30% and 90% shade treatments from all three blocks were collected at the time of final harvest and shipped to the Oak Ridge National Laboratory under cool and moist conditions and maintained within a greenhouse using the same neutral density shade cloth levels as used in the field study. All physiological measurements were completed within 48 h from field collection with a subset of plants measured at both the start and end of the study to ensure that photosynthetic responses did not change during the measurement period. *Sphagnum divinum* was not present on some of the hollow plots, and therefore are not included in this analysis. Carbon dioxide exchange and chlorophyll‐*a* fluorescence measurements were performed using a 6800‐18 aquatic chamber connected to an LI‐6800 open‐flow and steady‐state gas exchange system (LI‐COR Biosciences). The aquatic chamber is designed to allow for simultaneous measurements of chlorophyll‐*a* fluorescence and CO_2_ exchange from a liquid and partial liquid sample. The addition of water maintains plant water status, which is a major constraint for *Sphagnum*, and bryophytes in general, that do not have stomata. Measurements were performed using a reference CO_2_ at 400 μmol mol^−1^, a sample flow rate of 700 μmol s^−1^ and water vapor control at 20 mmol mol^−1^. For photosynthetic light response curves, approximately 10 stems were placed within the cuvette (capitulum plus 3 cm of stem) per sample. Samples were acclimated in the chamber for 15–20 min until steady‐state gas exchange and chlorophyll fluorescence were achieved. Measurements started with dark‐acclimated material and progressed with the following irradiance regime: 0 (dark respiration), 30, 75, 150, 250, 500, 750, 1000, 1250 μmol photons m^−2^ s^−1^. At each light level, gas exchange measurements were recorded following stabilization for at least 3 min. A_sat_ was the highest measured value from the light response curve. Fluorescence measurements were performed during the light response curves using a saturating light intensity of 10,000 μmol m^−2^ s^−1^ for 1000 ms. Steady‐state (F_s_) was measured at 50 kHz and maximum fluorescence (F_m_′) was measured at 250 kHz during the saturating flash.

### 2.6 Statistical analysis

Statistical analyses were conducted with Statistix 8.0 software (Analytical Software). *Sphagnum* growth data were initially analyzed as a randomized complete block split plot design, with three blocks and main plots within block comprising the 10 combinations of shade level and microtopography (hummock vs. hollow) and species within main plot as a split plot. If the microtopography and species effects were significant (*p* < .10), species response to shade in hummocks or hollows was analyzed by linear regression. If microtopography and species were not significant, polynomial contrasts of responses to shade across the pooled data were conducted. Data used in these analyses are freely available (Norby et al., 2023).

## 3 RESULTS

### 3.1 Light environment

Daily photosynthetic photon flux density (mol m^−2^ day^−1^), averaged across all days and the three replicate plots per treatment, was used to calculate the percentage shade relative to the open plots (Table 1). These measured values of light reduction integrated over the duration of the experiment were greater than the nominal values described by the shade cloth density. To provide perspective on the attained light levels relative to the light environment beneath shrubs in the SPRUCE chambers, HOBOs were deployed from 15 September to 15 October beneath heavy shrub shade (PAR = 0.24 mol m^−2^ day^−1^) and under moderate shade (PAR = 1.27 mol m^−2^ day^−1^). During this same time period, light under 80% shade cloth was 1.18 mol m^−2^ day^−1^ and under 90% shade cloth was 0.59 mol m^−2^ day^−1^. Hence, our shade treatments were realistic and encompassed the range of light quantity under the shrub cover in the SPRUCE enclosures. However, vegetative shade is known to alter light quality (e.g., red/far‐red ratio) that can influence plant growth in addition to reduced light quantity (Smith, 2000). Yet little is known about how light quality influences *Sphagnum* species or how the relative amount of shading from trees, shrubs, or herbaceous vegetation changes light spectra throughout the season. Therefore, our shading treatments focused on the use of neutral‐density light reduction filters.

### 3.2 Growth response

**FIGURE 3:**
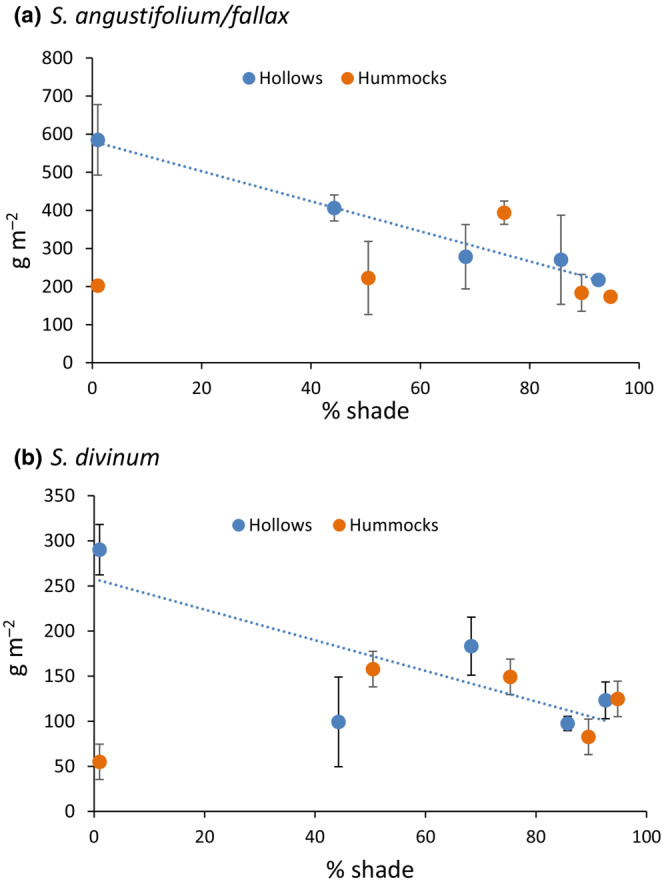
Dry matter increment of *Sphagnum* in response to shade. Data are the means of three blocks. (a) *S. angustifolium/fallax* in hummocks and hollows; in hollows: DW = −3.90 × %shade + 578, *r* ^2^ = .58, *p* = .001; (b) *S. divinum*; in hollows: DW = −1.68 × %shade + 257, *r* ^2^ = .44, *p* = .007.

Preliminary analysis of *Sphagnum* growth (dry matter accumulation) by analysis of variance indicated significant interactions among the effects of position (hummock or hollow), species, and shade cloth treatment. Hence, the response to the level of shade was analyzed separately for the four combinations of position and species within position. Growth of *S. angustifolium/fallax* in hollows declined linearly with increasing shade (*r* ^2^ = .58, *p* = .001), but there was no response to shade on the hummocks (Figure 3a). The difference in growth between hummocks and hollows occurred in the open plots and those with 30% shade cloth, where growth in hollows was about twice that on hummocks. The response of *S. divinum* was similar: a linear decline with increasing shade on hollows (*r* ^2^ = .44, *p* = .007), but not on hummocks (Figure 3b). Based on the regressions, there was a 60% growth reduction between open plots and the deepest shade for both species in hollows.

**FIGURE 4:**
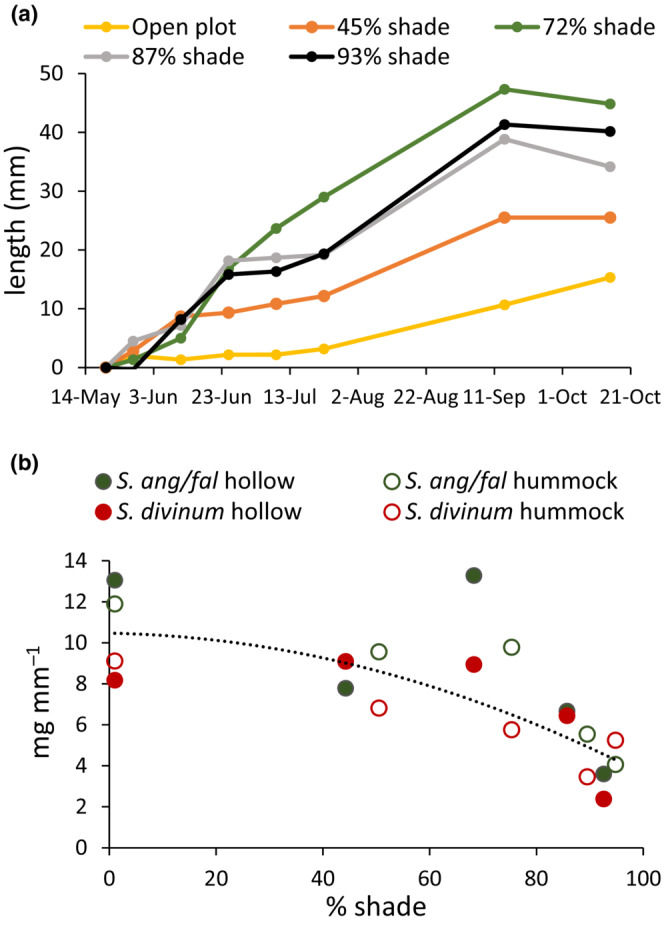
(a) New stem length in response to shade on hummocks, averaged over both in three blocks. Repeated measures analysis of variance indicated significant effects of shade, time, and shade × time interaction (*p* < .01). Effect of shade on final length was significant at *p* = .035. There was no effect in hollows. (b) Stem mass per unit length in October in response to shade. Mass per length = −0.0006 × (%shade)^2^–0.0045 × %shade +10.469; *r* ^2^ = .665.

*Sphagnum* height growth in the shade plots was monitored throughout the experiment. In contrast to dry matter increment, height growth was greater in the deeper shade treatments (Figure 4a). There was very little height growth in July and August when rainfall was especially sparse, and the *Sphagnum* was too dry and fragile to measure during the period. The different responses of height growth and dry matter increment are not contradictory; the discrepancy is explained by the significant decline in stem mass per unit length with increasing shade (Figure 4b). The *Sphagnum* stems in deep shade were etiolated—thin and spindly.

### 3.3 Evaluation of possible artifacts

**FIGURE 5:**
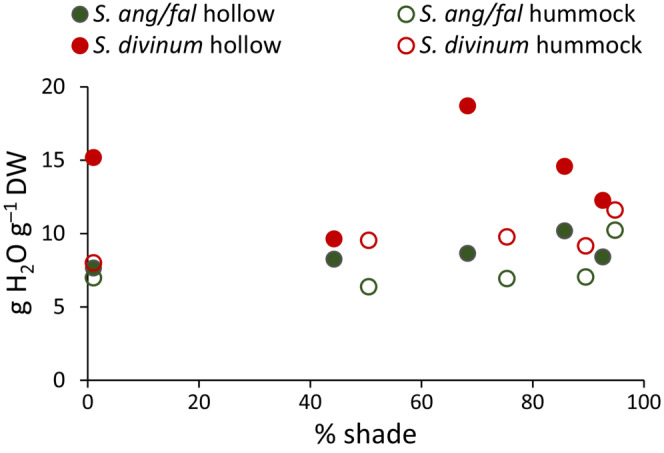
Water content of *Sphagnum* in October in response to shade. Data are the means of three replicates. There are no significant differences.

The shade cloths might have altered moisture conditions in addition to their direct effect on light. However, we saw no indication of this possible confounding effect: *Sphagnum* water content did not differ across shade treatments any time during the experiment, except measurements were not possible during the August drought. At the end of the experiment in October average water content was 10.0 g water per gram dry mass and similar across shade treatments (Figure 5). Surface temperatures, measured on three dates in June and July, were unaffected by shade cloth.

**FIGURE 6:**
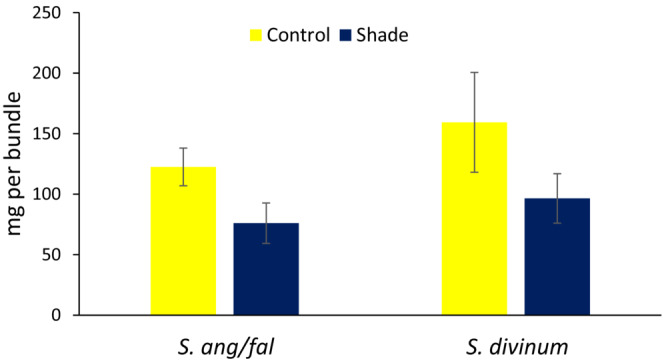
Dry weight increment of 10 *Sphagnum* stems in bundles (Figure 2c) under small cover of 90% shade cloth (Figure 2d) or control bundles with no shade cloth; shrubs were not removed. Data are the means over hollow and hummock locations in three blocks. Statistical significant for *S. angustifolia/fallax*: *p* = .068*; S. divinum*: *p* = .202.

Removal of shrubs from the shade plots might have had unintended effects on moisture conditions. However, when measured under intact shrub cover the relative decline of dry matter increment of *Sphagnum* bundles under small patches of 90% shade cloth versus unshaded bundles was similar to the decline in deep shade in the main shade experimental plots after shrub removal (Figure 6).

### 3.4 Shrub–litter interactions

**FIGURE 7:**
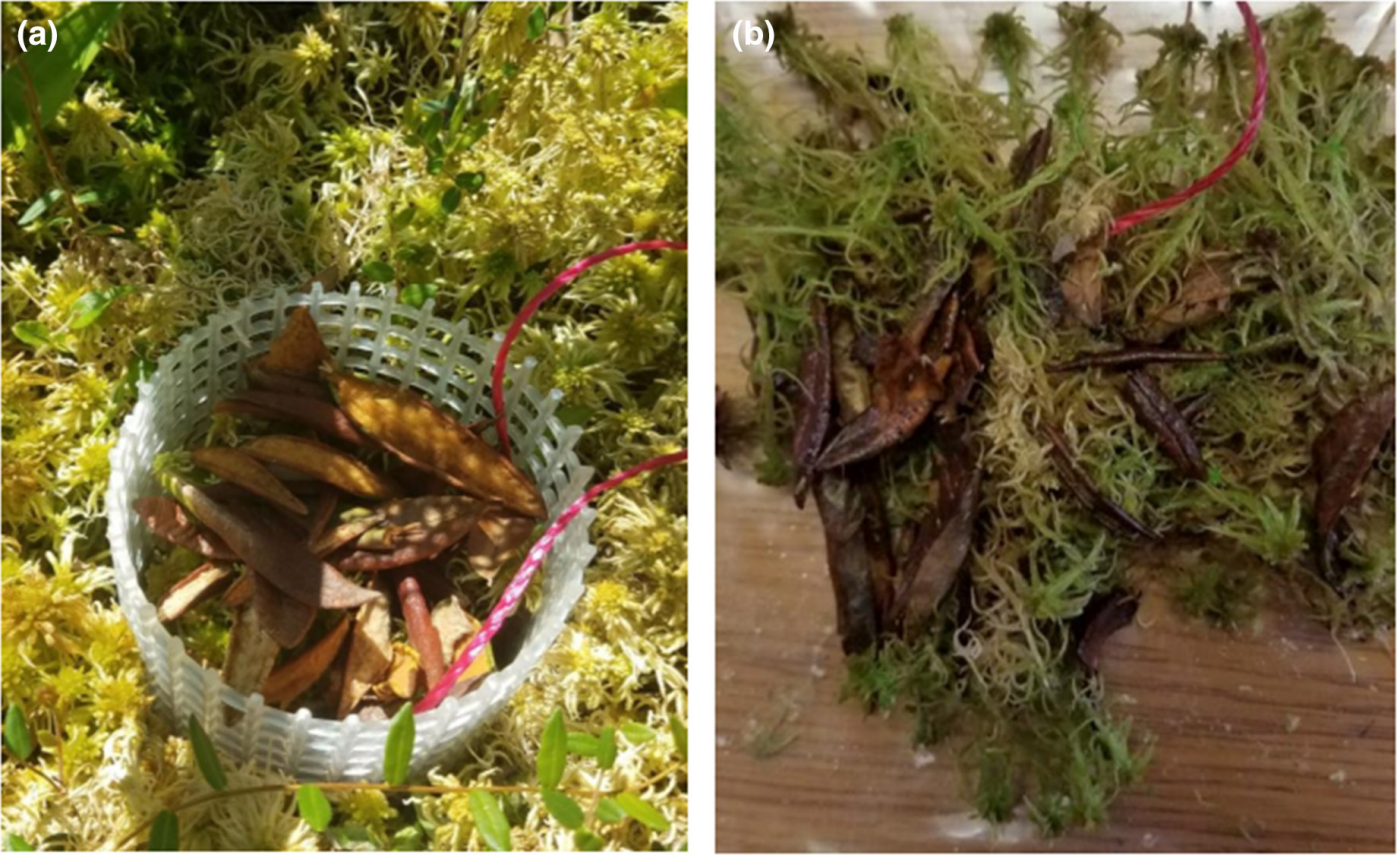
(a) Shrub litter added to top of *Sphagnum* in June, corresponding to amount of litter shown in Figure 2b. (b) Harvested *Sphagnum* in October showing interspersed litter throughout the new growth.

The dense mat of shrub litter that was placed on top of a column of *Sphagnum* in July (Figure 7a) became interspersed with the *Sphagnum* when they were harvested in October (Figure 7b). There was no apparent effect on *Sphagnum* growth and no difference in Ca or Mg concentrations (Table 2) or other macro‐ or micronutrients in *Sphagnum* capitula grown with or without litter addition.

**TABLE 2:** Calcium and magnesium concentrations in capitula of *Sphagnum* growing beneath different levels of shade and with added shrub litter or without (control).

% shade	Ca (mg g^−1^)	Ca (mg g^−1^)	Mg (mg g^−1^)	Mg (mg g^−1^)
	Control	+litter	Control	+litter
0	3.339 ± 0.225	4.315 ± 0.429	0.947 ± 0.019	1.303 ± 0.115
47.9	3.712 ± 0.449	2.128 ± 1.801	1.083 ± 0.037	0.790 ± 0.393
72.4	2.804 ± 0.714	3.289 ± 1.033	0.897 ± 0.104	1.283 ± 0.156
87.9	3.984 ± 0.518	3.583	1.237 ± 0.032	1.312
93.9	4.175 ± 0.967	3.558 ± 0.074	1.521 ± 0.205	1.278 ± 0.067
Mean	3.603 ± 0.200	3.375 ± 0.370	1.137 ± 0.070	1.193 ± 0.120

*Note*: Data are the means of hummock and hollow locations in each of three blocks ± SE.

### 3.5 Photosynthesis

**FIGURE 8:**
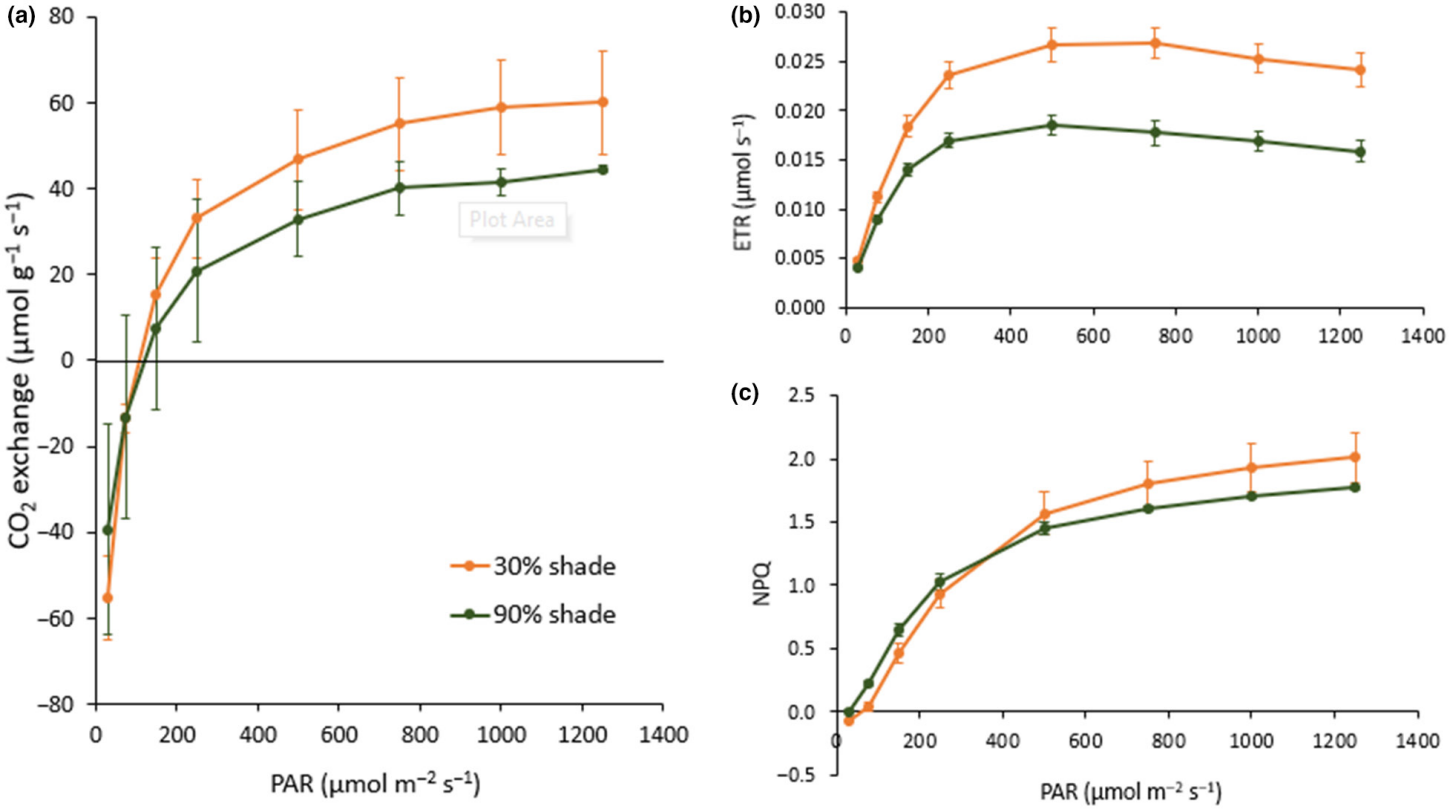
Photosynthetic light response curves of *Sphagnum angustifolium/fallax* from hollows and grown under 30% or 90% shade cloth. (a) CO_2_ exchange rate, (b) electron transport rate (ETR), and (c) non‐photosynthetic chlorophyll fluorescence quenching (NPQ). Data are the means of three replicate samples of 10 stems ± SE. Differences between shade levels at PAR = 1250 μmol m^−2^ s^−1^ were significant at *p* = .071 for CO_2_ exchange rate, *p* = .0412 for ETR, and *p* = .001 for NPQ.

Leaf light response curves indicate that maximal light saturated (*A*_sat_) photosynthesis was 42% greater for *S. angustifolium/fallax* grown under 30% shade cloth relative to plants grown under 90% shade cloth (Figure 8). The estimated proportion of electrons passing through PSII (ETR) as measured by chlorophyll‐*a* fluorescence showed a similar, yet slightly more exaggerated trend than *A*_sat_, with a 51% maximum difference between 30% and 90% shade cloth treatments. Non‐photochemical quenching (NPQ) again showed a similar trend as *A*_sat_ and ETR indicating that the greater photosynthetic capacity of plants grown under 30% shade cloth also had greater photoprotection via enhanced thermal dissipation of excess excitation energy in PSII. Relative to seed plants, dark respiration for these *Sphagnum* samples is higher than expected and likely a result of high microbial biomass from the *Sphagnum*‐associated microbiome. *Sphagnum* plants contain upward of 70% of their cells as dead hyaline cells that hold water and host their associated microbiome (Carrell et al., 2022; Kostka et al., 2016). This enhanced microbial biomass with resulting microbial respiration would increase our dark respiration values.

## 4 DISCUSSION

The response of *Sphagnum* growth in response to increasing shade is consistent with the hypothesis that increased shade resulting from shrub expansion in the warmer SPRUCE chambers contributed to reduced *Sphagnum* growth. Growth loss due to increased shading in hollows was 60%, in support of hypothesis 1. Shading from shrubs in the SPRUCE experiment may have additional effects through changes in light quality, which were not addressed in this experiment. Small and inconsistent effects of light quality (e.g., red/far‐red ratio) on morphology of bryophytes have been noted in several experiments (Hoddinott & Bain, 1979; van der Hoeven et al., 1998; Vicherova et al., 2020).

The response to in situ shading was consistent with observations from other experiments conducted in mesocosms and greenhouse conditions. Biomass production of *S. capillifolium* in mesocosms was 35% less when maintained at a photosynthetic photon flux density (PPFD) of less than 40 μmol m^−2^ s^−1^ compared to controls at PPFD > 300 μmol m^−2^ s^−1^ (Bonnett et al., 2010). In our study, the average daytime PPFD was <40 μmol m^−2^ s^−1^ in the deepest shade and was >300 μmol m^−2^ s^−1^ only in the open plots. However, shading to 40 μmol m^−2^ s^−1^ in growth chambers had no effect on biomass production of *S. capillifolium*, *S. palustre*, and *S. fallax* (Ma et al., 2015).

Field studies also have suggested an effect of shading from shrubs on *Sphagnum* productivity. At the Mer Bleue bog in Canada, increased N fertilization led to increased shrub cover, which reduced PAR reaching the peat surface to 75 μmol m^−2^ s^−1^, or 23% of the PAR in unfertilized plots. Although the denser shrub cover also cooled the surface soil, the effects of temperature and moisture on *Sphagnum* were small compared to the influence of light in an associated greenhouse study. Statistical analysis supported the conclusion that the absence of moss in fertilized plots might have been due to decreased light availability (Chong et al., 2012), although modeling studies of the site suggest that N toxicity to *Sphagnum* could not be excluded (Wu & Blodau, 2015). Other observations in field studies also have demonstrated an effect of shrub biomass on moss production (Malmer et al., 2003; Murray et al., 1993) and the reduction of light beneath the shrub cover (Bubier et al., 2007; Juutinen et al., 2010; Limpens et al., 2003). If light is assumed to be a limiting resource for *Sphagnum* (Chapin & Shaver, 1985; Kuiper et al., 2014; van der Wal et al., 2005), the common inference is that shading affects *Sphagnum* productivity through reduction in light, although other effects may also be important, such as reduction in soil moisture (Harley et al., 1989). Potvin et al. (2015), however, reported no effect of shrub removal on *Sphagnum* productivity in a mesocosm study, but no data on the light environment were presented. None of these studies included controlled shading and the isolation of light reduction from other potential effects of shrubs on *Sphagnum*. In the SPRUCE chambers, shrubs might well have affected *Sphagnum* productivity through drawdown of water, especially in combination with warming treatments, but in the current shade experiment, shrubs were excluded and there were no effects of shade cloth on *Sphagnum* moisture.

The decline in growth (dry matter increment) with increasing shade was observed only in hollows; growth in hummocks was unresponsive to shade treatments. On hummocks, shading led to increased stem length, which was compensated by decreased mass per unit length. The difference in response between hummocks and hollows occurred at the two brightest treatments where the average daily PPFD and the PPFD at solar noon were greater on hummocks than in hollows (Table 1). It may be that the higher light levels are inhibitory to *Sphagnum* photosynthesis and growth as made apparent in our study with reduced ETR at high light. Bryophytes are generally considered to be shade‐adapted plants, reaching photosynthetic light saturation at low irradiances between 30 and 300 μmol m^−2^ s^−1^ (Bonnett et al., 2010; Davey & Rothery, 1997). In this study, light saturation was within 9% of maximum values by 750 μmol m^−2^ s^−1^ PAR, and light compensation point was at 100 μmol m^−2^ s^−1^. During periods of bright, dry, sunny weather bryophytes will generally be dry and metabolically inactive; photosynthesis occurs mostly in rainy or cloudy weather, when irradiance may often be <20% of full sunlight (Bonnett et al., 2010). Vascular plants at lower densities reduce evaporation rates (Heijmans et al., 2001; Rincon & Grime, 1989; van der Wal et al., 2005) and may therefore be beneficial to *Sphagnum* species by preventing capitulum desiccation (Ma et al., 2015; Marschall & Proctor, 2004).

The dramatic decline in *Sphagnum* NPP in the SPRUCE enclosures was strongly associated with increased air and soil temperature and drier conditions (Norby et al., 2019) and was primarily associated with the loss of *Sphagnum* cover rather than the decline in dry matter increment of living *Sphagnum*. It is reasonable to suggest that growth loss and etiolation associated with shading made the *Sphagnum* community more vulnerable to the deleterious effects of hot and dry conditions, and these effects were cumulative over time contributing to mortality. Etiolation is a common response of *Sphagnum* to shading (Bengtsson et al., 2016; Hayward & Clymo, 1983). In the experiment of Ma et al. (2015), height increment increased with shading although there was no response of *Sphagnum* biomass.

In the SPRUCE experiment, some spots with no live *Sphagnum* cover in the warmest chambers were covered with a dense mat of leaf litter from shrubs (Figure S2b). It was unclear whether the litter accumulation was a cause of *Sphagnum* decline, for example by completely blocking light, or rather occurred only after the *Sphagnum* had declined. In the shade plots, the mat of shrub litter that we added to match the amount observed in the warmest SPRUCE chambers (Figure 7a) had no apparent effect on the *Sphagnum* beneath it. The *Sphagnum* grew through the litter mat, and at the end of the experiment, the litter was dispersed through the column of *Sphagnum* (Figure 7b). Hence, we conclude that shrub litter accumulation was unlikely to be a direct cause of *Sphagnum* death. Leaf litter from *Betula neoalaskana* (paper birch) reduced growth of feather moss in an Alaskan boreal forest, which was likely associated with physical effects such as shading and crushing (Jean et al., 2020), but we note that birch leaves are much larger than the shrub litter in our experiment. Leachate of birch litter had no effect on feather moss. We investigated the possibility that elements leaching from shrub litter had inhibitory effects on *Sphagnum* growth. High Ca^2+^ concentrations can be detrimental to *Sphagnum* (Clymo, 1973; Vicherova et al., 2015), but this interaction occurs primarily in minerotrophic sites with high pH and is unlikely in acid bogs such as the SPRUCE site (Koks et al., 2019). Typical bog species and poor fen species were little affected by submersion in high Ca^2+^ solutions (Koks et al., 2022). Analysis of Ca^2+^, Mg^2+^, and other element concentrations in *Sphagnum* grown with or without added shrub litter indicated no effect of the litter on element concentrations in *Sphagnum*, and we conclude that physical or chemical interactions with shrub litter did not contribute to *Sphagnum* decline in the SPRUCE experiment. However, we cannot exclude the possibility that chemical interactions might have developed over a longer term as the shrub litter decomposed.

The *Sphagnum* community will be a critical determinant of peatland responses to climatic change, and Earth system models are now incorporating *Sphagnum* processes to improve predictive assessments of peatlands under environmental change (Shi et al., 2021). Disentangling the direct and indirect effects on *Sphagnum* should inform how best to represent *Sphagnum* in models. This experiment has demonstrated that the indirect effect of shrub expansion and the attendant increase in shade in response to warming may have contributed to the decline of *Sphagnum* in the SPRUCE experiment and can be presumed to be a factor in similar peatland ecosystems in response to climatic change.

## AUTHOR CONTRIBUTIONS

**Richard J. Norby:** Conceptualization (equal); formal analysis (lead); investigation (equal); methodology (lead); writing – original draft (lead). **Taylor Baxter:** Investigation (equal); methodology (supporting); writing – review and editing (supporting). **Tatjana Živković:** Conceptualization (equal); methodology (supporting); writing – review and editing (supporting). **David J. Weston:** Conceptualization (equal); formal analysis (equal); investigation (equal); methodology (equal); writing – original draft (supporting).

## CONFLICT OF INTEREST STATEMENT

None declared.

## DECLARATION

This manuscript has been authored by UT‐Battelle, LLC under Contract No. DE‐AC05‐00OR22725 with the U.S. Department of Energy. The United States Government retains and the publisher, by accepting the article for publication, acknowledges that the United States Government retains a non‐exclusive, paid‐up, irrevocable, worldwide license to publish or reproduce the published form of this manuscript, or allow others to do so, for United States Government purposes. The Department of Energy will provide public access to these results of federally sponsored research in accordance with the DOE Public Access Plan (https://energy.gov/doe‐public‐access‐plan).

## Supporting information


Appendix S1

## Data Availability

Data are freely available from the SPRUCE project data archive: https://doi.org/10.25581/spruce.049/1426474 and https://doi.org/10.25581/spruce.106/1961626.
