# Supplementary material for: Shading contributes to Sphagnum decline in response to warming
Source: Ecol Evol. 2023 Sep 19;13(9):e10542. doi: 10.1002/ece3.10542 (PMC10507575; doi:10.1002/ece3.10542)
Supplement: Supplementary file 1 — Appendix S1 [file ECE3-13-e10542-s001.docx]

**Appendix S1**

**Description of SPRUCE experiment manipulations**

Climate change manipulations were established in 10 large, open-top enclosures described by Hanson et al. (2017). The experimental plots are within 12.8-m diameter x 7-m tall octagonal enclosures sheathed with double-walled transparent greenhouse panels and are hydrologically isolated from the surrounding bog by a subsurface corral (Hanson et al., 2017; Sebestyen & Griffiths, 2016). Air warming is achieved with propane-fired heat exchangers and a system of blowers and conduits (Hanson et al., 2017) with target values of +0, +2.25, +4.5, +6.75, and +9°C; the +0°C enclosures are generally 1 to 2°C warmer than outside ambient air. Soil (peat) warming is achieved with a belowground heating array of 3-m vertical low-wattage heating elements installed within plastic-coated iron pipe (Hanson et al., 2017). For analyses of *Sphagnum* responses, the warming treatments are characterized by the temperature 0.5 m above the surface over the period from 15 April to 15 October. One of the two enclosures at each temperature began to receive elevated CO_2_ on 15 June 2016 to approach concentrations of 500 ppm greater than ambient, or about 900 ppm (Hanson et al., 2017).

***Sphagnum* productivity, 2019-2021**

Measurements of *Sphagnum* productivity, as previously reported through 2018 (Norby et al., 2019), continued in 2019-2021. The protocol as fully described in Norby et al. (2019) was followed without alteration. Briefly, annual growth of *S. angustifolium/fallax* in hummocks and hollows and *S. divinum* in hummocks was measured in mesh columns that were installed in each of the 10 treatment enclosures in October and retrieved one year later. Fractional cover of the two *Sphagnum* species, other bryophytes, dead *Sphagnum*, or bare ground was recorded annually in October at 25 5 x 5 cm locations along three transects within each enclosure. *Sphagnum* NPP was calculated as dry matter increment per unit area multiplied by the fractional cover and reported in grams C per square meter per year based on the measured *Sphagnum* tissue C fraction of 0.429.

The response of *Sphagnum* productivity to warming and CO_2_ enrichment in 2019-2021 (Figure S1a) was very similar to that observed in 2018. NPP declined linearly with increasing temperature, and there was a significant interaction with CO2 such that NPP was lower in elevated CO2 in the cooler enclosures. There was little response to the treatments in the first year of exposure (2016), but the response became apparent in the second and third years, with little change after that (Figure S1b,c). The loss of cover was the primary contributor to the decline in NPP (Norby et al. 2019; Petro et al. 2023). Data used in these analyses are freely available (Norby & Childs 2018).

There was a dense cover of shrubs in the warmest chambers (Figure S2a), and shrub litter accumulated in dense mats (Figure S2b). Spot measurements of PAR above and below shrubs were used to provide guidance for the levels of shade treatment to be used in the shade experiment (Figure S3).

**Figure S1.** Net primary production of *Sphagnum* in the experimental enclosures. (a) Responses to temperature in 2021; ambient CO_2_: NPP = -14.17 × T + 367, r^2^ = 0.764, p= 0.052; elevated CO_2_: NPP = -7.87 × T + 205, r^2^ = 0.749, p=0.058. (b) Time series of NPP response to warming under ambient CO_2_. (c) Time series under elevated CO_2_.


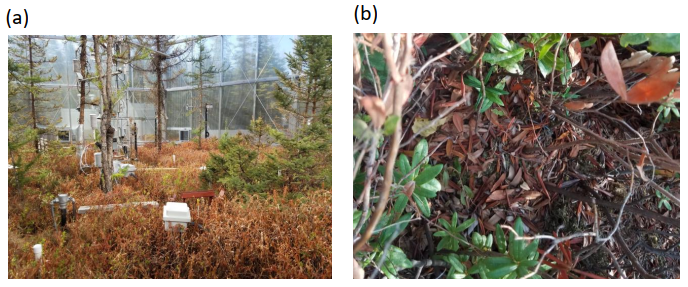


**Figure S2.** (a) Dense shrub layer in SPRUCE enclosure at +9 °C; (b) Shrub litter over ground with no *Sphagnum* in +9 °C SPRUCE enclosure

**
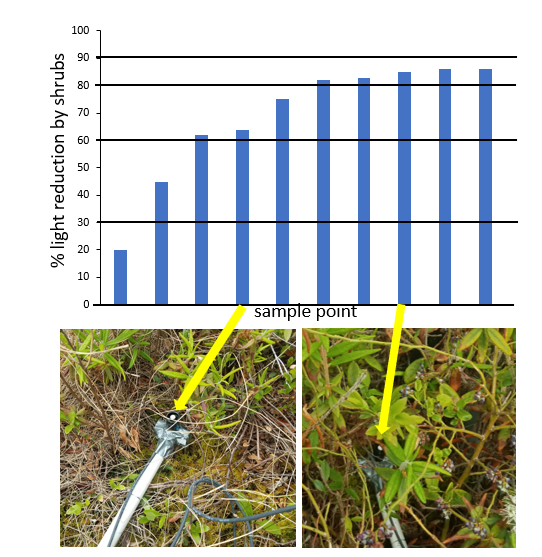
**

Figure S3. Percentage reduction in PAR (µmol m^-2^ s^-1^) beneath shrubs relative to PAR immediately above the shrub layer. Measurements were made at 10 sample points within a SPRUCE enclosure with the +9 °C warming treatment. The solid horizontal lines indicate the nominal shade levels chosen for the shade experiment. The photos shows the shrub cover at sample points with 64% shade and 83% shade; arrows point to the PAR sensor.

**References for Appendix**

Hanson, P. J., Riggs, J. S., Nettles, W. R., Phillips, J. R., Krassovski, M. B., Hook, L. A., . . . Barbier, C. (2017). Attaining whole-ecosystem warming using air and deep-soil heating methods with an elevated CO_2_ atmosphere. *Biogeosciences, 14*(4), 861-883. doi:10.5194/bg-14-861-2017

Norby, R. J., Childs, J., Hanson, P. J., & Warren, J. M. (2019). Rapid loss of an ecosystem engineer: *Sphagnum* decline in an experimentally warmed bog. *Ecology and Evolution, 9*(22), 12571-12585. doi:10.1002/ece3.5722

Norby, R. J., & Childs, J. (2018). SPRUCE: *Sphagnum* Productivity and Community Composition in the SPRUCE Experimental Plots. Oak Ridge National Laboratory, TES SFA, U.S. Department of Energy, Oak Ridge, Tennessee, U. S. A. https://doi.org/10.25581/spruce.049/1426474

Petro, C., Carrell, A., Wilson, R. M., Duchesneau,K. Noble-Kuchera, S., Song, T.,…. Kostka, J. E. (2023). Climate drivers alter nitrogen availability in surface peat and decouple N_2_ fixation from CH_4_ oxidation in the *Sphagnum* moss microbiome. *Global Change Biology, 29(11), 3159-3176. doi:*doi.org/10.1111/gcb.16651

Sebestyen, S. D., & Griffiths, N. A. (2016). SPRUCE enclosure corral and sump system: Description, operation, and calibration. Publication no. 10.3334/CDIAC/spruce.030.
